# First molecular evidence of potential *Culicoides* vectors implicated in bluetongue virus transmission in Morocco

**DOI:** 10.1186/s13071-024-06167-y

**Published:** 2024-02-19

**Authors:** Soukaina Daif, Ikhlass El Berbri, Ouafaa Fassi Fihri

**Affiliations:** Microbiology, Immunology, and Infectious Diseases Unit, Department of Pathology and Veterinary Public Health, Institut Agronomique et Vétérinaire Hassan II, Rabat, Morocco

**Keywords:** Bluetongue, *Culicoides*, RT-qPCR, Potential vectors, MIR, MLE, Season, Morocco

## Abstract

**Background:**

Bluetongue is a non-contagious viral disease that affects both domestic and wild ruminants. It is transmitted primarily by small hematophagous Diptera belonging to the genus *Culicoides* (Diptera: Ceratopogonidae). The current study represents the first molecular investigation into the potential role of *Culicoides imicola*, *Culicoides paolae*, *Culicoides newsteadi*, *Culicoides* spp., and *Culicoides circumscriptus* as bluetongue virus (BTV) vectors in Morocco. Additionally, the study aimed to evaluate the vectorial activity of midges during the survey seasons.

**Methods:**

Parous females of these species were captured from several regions of Morocco (6 out of 12) from 2018 to 2021 using Onderstepoort Veterinary Institute (OVI) traps. A total of 2003 parous female specimens were grouped into 55 batches. The midge body of each batch was dissected into three regions (head, thorax, and abdomen), and these regions were analyzed separately using reverse transcription quantitative polymerase chain reaction (RT-qPCR).

**Results:**

BTV RNA was detected in 45 out of the 55 batches tested, indicating a positivity rate of 81.8%. The RT-qPCR-positive pools of the studied *Culicoides* species exhibited high levels of BTV positivity in each body part (head, thorax, and abdomen), confirming the successful replication of the virus within midge bodies. The BTV circulation was substantial across all three survey seasons (spring, summer, and autumn). High infection rates, calculated using the minimum infection rate (MIR) and maximum likelihood estimation (MLE), were observed during the collection seasons, particularly in autumn and spring, and for all investigated *Culicoides* species, most notably for *C. imicola* and *C. newsteadi*. These increased infection rates underscore the significant risk of *Culicoides* transmitting the BTV in Morocco.

**Conclusions:**

The detection of BTV positivity in *Culicoides* spp. (lacking wing spots that allow their differentiation according to morphological identification keys) suggested that other *Culicoides* species are competent for BTV transmission in Morocco. The study results indicated, for the first time at the molecular level, that *C. imicola* and *C. newsteadi *are the primary potential vectors of BTV in Morocco and that *C. paolae* and *C. circumscriptus* are strongly implicated in the propagation of bluetongue at the national level.

**Graphical Abstract:**

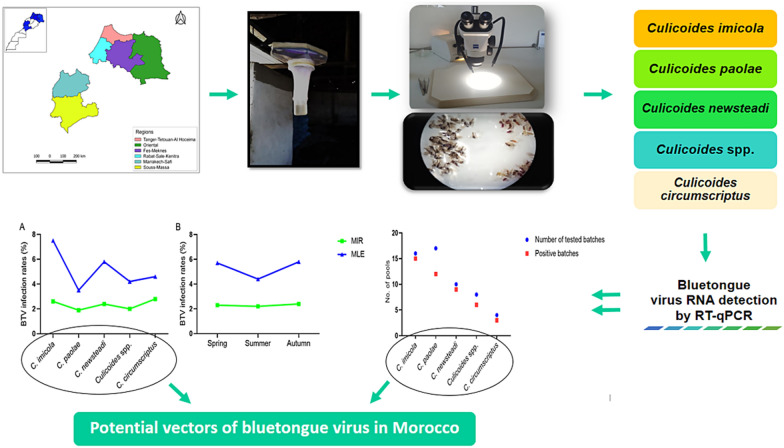

## Background

Bluetongue disease is a non-contagious viral disease affecting domestic and wild ruminants, and it is subject to mandatory notification to the World Organization for Animal Health (WOAH). It is primarily transmitted through *Culicoides* bites during a blood meal, resulting in significant economic losses [1]. Bluetongue virus (BTV) belongs to the *Orbivirus* genus of the Reoviridae family, characterized by a double-stranded RNA genome with 10 segments coding for various proteins. Currently, at least 36 different BTV serotypes are circulating worldwide [2–4].

Bluetongue first emerged in Morocco in 1956 and reappeared in 2004, triggering a severe epizootic. Since then, the disease has become enzootic, with several outbreaks reported annually throughout the country involving various serotypes [5–9]. Previous entomological investigations in Morocco have identified 56 *Culicoides* species, including at least eight species that are the principal or potential vectors of animal viruses [10–17].

A series of events occurs in female midges following ingestion of the virus during their blood meal. These include the liberation of the virus, followed by the infection of the midgut cells, proliferation within these cells, escape from the midgut, spreading into extraintestinal tissues, infection of the salivary glands located between the head and thorax, and finally, transmission of the virus to the subsequent host through the salivary glands. The viral particles must break through various barriers to infection within the abdomen, thorax, and head to successfully disseminate throughout the insect body. These obstacles influence the virus titer and its prevalence during infection, impacting pathogen transmission rates during feeding events. Moreover, the insect's susceptibility or refractoriness to a virus is related to extrinsic factors such as gut microbiota, as well as heritable genetic features like antiviral immune defenses in the insect. These factors are crucial for understanding the vector competence of a midge [18–23].

Detection, quantification, and isolation of the virus in field-collected *Culicoides* midges, analysis of infection levels under natural conditions, and laboratory infection trials are methods for assessing the vectorial competence of *Culicoides*. The species vectors of BTV have been determined accordingly [24–26]. Approximately 30 *Culicoides* species have been associated with BTV transmission, among which *C. imicola* is recognized as the major vector in Africa [27, 28]. Due to challenges in conducting studies on *Culicoides* vector competency, natural infection rates are generally considered a reliable approximation of the vector role [26].

The endemic status of bluetongue in Morocco underscores the importance of identifying *Culicoides* species vectors of the disease to implement targeted management strategies. Consequently, assessing the infection levels of these species is imperative for a deeper understanding of the transmission dynamics of the disease.

The current study marks the first molecular investigation in Morocco to investigate the potential role of *Culicoides imicola*, *Culicoides paolae*, *Culicoides newsteadi*, *Culicoides* spp., and *Culicoides circumscriptus*, collected during entomologic investigations conducted between 2018 and 2021 in different Moroccan regions. The study aimed to assess their involvement in BTV transmission, explore vectorial activity seasons, and investigate their epidemiological contribution and vectorial competence. This was achieved through the detection of virus dissemination in the bodies of field-caught parous females of these species, and the examination of infection rates using two estimates, the minimum infection rate (MIR) and the maximum likelihood estimation (MLE).

## Methods

### *Culicoides* field collection

**Fig. 1:**
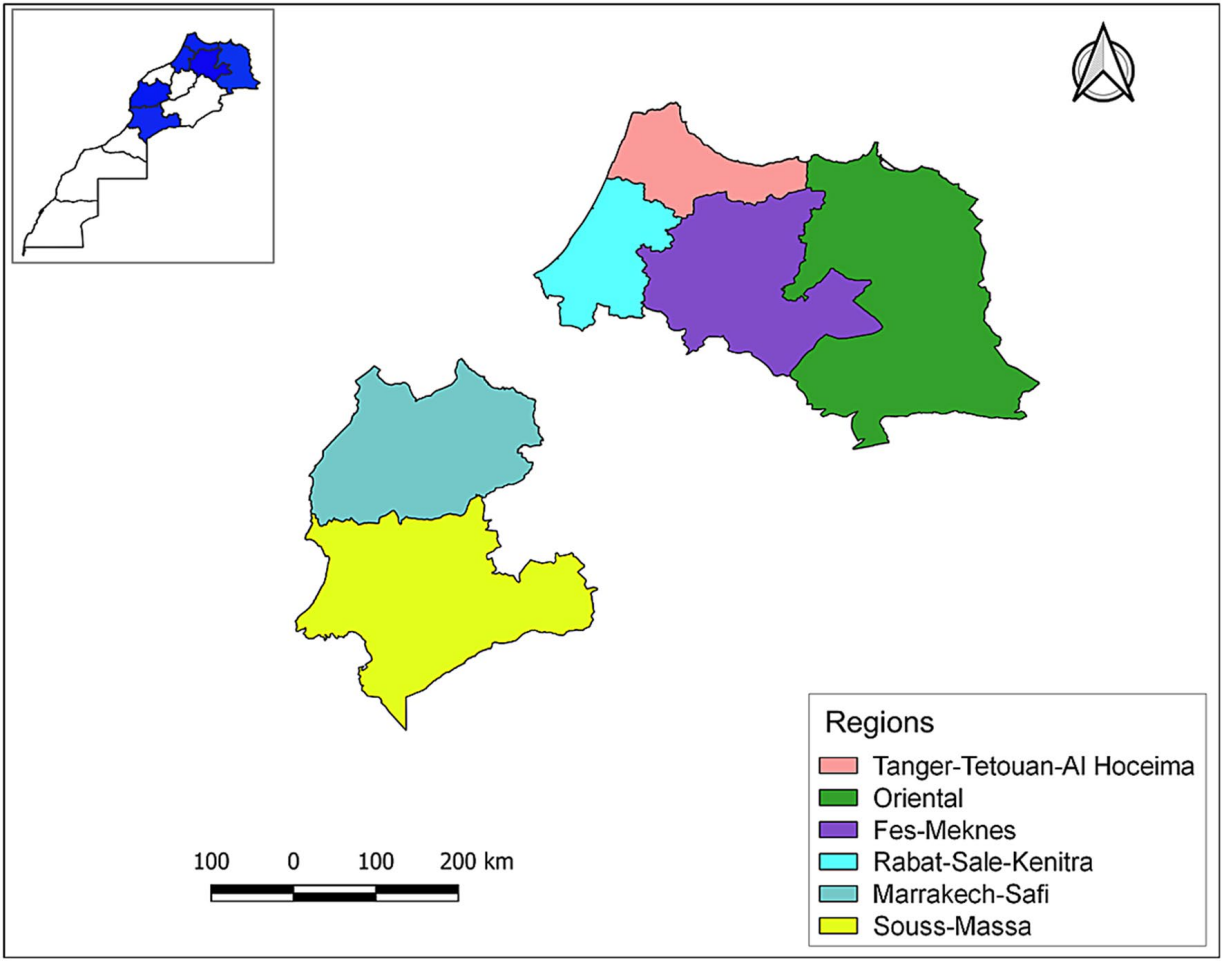
Regions of origin for *Culicoides* species investigated in the Kingdom of Morocco from 2018 to 2021

Parous females of *C. imicola*, *C. paolae*, *C. newsteadi*, *Culicoides* spp., and *C. circumscriptus* were collected using Onderstepoort Veterinary Institute (OVI) light traps across different regions in Morocco (6 out of 12) from 2018 to 2021 (Fig. 1). Species were identified using relevant morphological keys [29, 30]. The designation *Culicoides* spp. is assigned to those lacking wing spots, aiding differentiation according to identification keys. The midges were collected in a solution specifically prepared in the laboratory, composed of ethylenediaminetetraacetic acid (EDTA), sodium citrate, ammonium sulfate, and sterile water. This solution was designed to preserve any potential viral RNA that could be present in *Culicoides* at ambient temperature during the night of capture, from sunset to sunrise. Subsequently, the *Culicoides* were transferred to 70% ethanol and stored at −20 °C.

### Detection of BTV infection in *Culicoides* midges

A total of 2003 specimens were grouped into 55 pools, each containing no more than 45 midges from the same zone and catch time.

The midges were dissected and separated into three parts: head, thorax, and abdomen. Each body region was collected in a separate subpool. Thus, every pool was subdivided into three subpools.

Viral RNA was isolated from the supernatant obtained by grinding the insect pools in phosphate-buffered saline (PBS) using the MagMAX™ Viral RNA Isolation Kit (Applied Biosystems) according to the manufacturer's instructions.

Reverse transcription quantitative polymerase chain reaction (RT-qPCR) assays were performed using the LSI VetMAX™ BTV NS3 All Genotypes Kit (Applied Biosystems) in accordance with the manufacturer's instructions. The kit includes two sets of primers/TaqMan probes, one labeled FAM™–NFQ (non-fluorescent quencher), encoding segment 10 of BTV RNA, and the other labeled VIC™–TAMRA™, for the internal positive control.

Reverse transcription and amplification were performed on an Applied Biosystems 7500 Fast Real-Time PCR system with the following thermal cycler program: 45 °C for 10 min, 95 °C for 10 min, followed by 40 cycles of denaturation (95 °C for 15 s) and annealing/extension (60 °C for 45 s). The cycle threshold (Ct) value was defined once the fluorescent signal breached a threshold fluorescence line. An insect pool with a Ct lower than 40 on the FAM-NFQ detector was considered BTV-positive.

### Data processing and statistical analysis

QGIS 3.30.1 software was used to construct a map showing the regions of origin of the studied *Culicoides* species in Morocco.

The data were analyzed using R software (version 4.2.3). Infection rates for *Culicoides* were calculated using the *PooledInfRate* package, with a confidence interval (CI) of 95% and a scale of 100. MLE estimate infection rates based on probabilistic models following a binomial distribution. The MIR defines the minimum number of infected individuals per 100 midges tested. Pearson’s Chi-square and Fisher’s exact tests were used to compare *Culicoides* pool positivity rates by species and season. The simultaneous application of both tests strengthened the validity of the results. Differences were considered statistically significant at *P* < 0.05 and a 95% CI.

## Results

### RT-qPCR results for *Culicoides* batches

The individual analysis of the three body regions of the specimens within each pool revealed similar results. The Ct values of the head, thorax, and abdomen subpools were similar for each origin pool (Table 1). Consequently, RT-qPCR results are presented based on the pools of origin.

**Table 1:** Average Ct values of various body parts in parous *Culicoides* females collected in Morocco (6 out of 12 regions) from 2018 to 2021

Species	RT-qPCR mean Ct values		
	Head	Thorax	Abdomen
*C. imicola*	24.3	24.1	24.6
*C. paolae*	26.3	26.8	26.5
*C. newsteadi*	27.2	26.9	27.5
*Culicoides* spp.	25.8	25.2	25.6
*C. circumscriptus*	26.6	26.1	26.4

*Ct* Cycle threshold

**Fig. 2:**
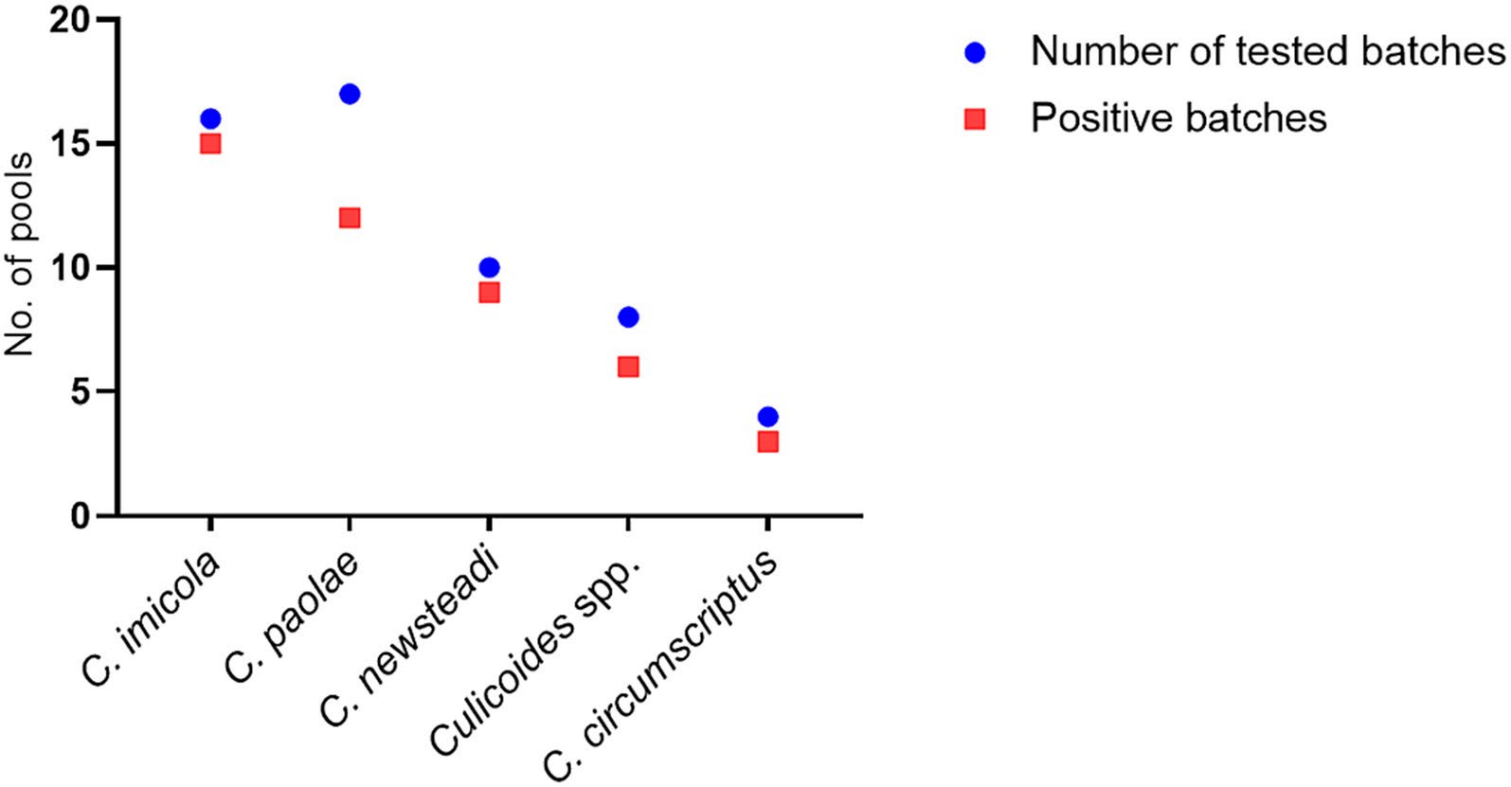
Batches of *Culicoides* species tested for bluetongue virus by RT-qPCR

Among the 55 *Culicoides* batches screened for BTV, 45 tested positive (81.8%). BTV was detected in 15 of 16 batches of *C. imicola* (93.7%), 12 of 17 of *C. paolae* (70.6%), 9 of 10 of *C. newsteadi* (90%), six of eight of *Culicoides* spp. (75%), and three of four batches tested of *C. circumscriptus* (75%) (Fig. 2).

The statistical analysis showed no significant differences among batches of different species (Chi-square test, *χ* 2 = 3.797, *df* = 4, *P* = 0.434; Fisherʼs exact test, *P* = 0.374).

The viral loads of positive pools were significant for all tested species, with Ct < 30. Ct values for positive pools of *C. imicola* ranged between 19.6 and 29.5, *C. paolae* between 24.7 and 29, *C. newsteadi* between 18.4 and 29.8, *Culicoides* spp. between 22 and 25.8, and *C. circumscriptus* between 21.1 and 26.6. The highest virus loads were observed in the head, thorax, and abdomen of *C. imicola* and *C. newsteadi* (Ct < 20) (Table 1).

### RT-qPCR results for *Culicoides* pools according to season

The different batches comprised specimens collected during three seasons (spring, summer, and autumn). The analysis resulted in nine *Culicoides*-positive pools out of 10 collected in spring (90%), 25 out of 32 in summer (78.1%), and 11 out of 13 in autumn (84.6%).

**Fig. 3:**
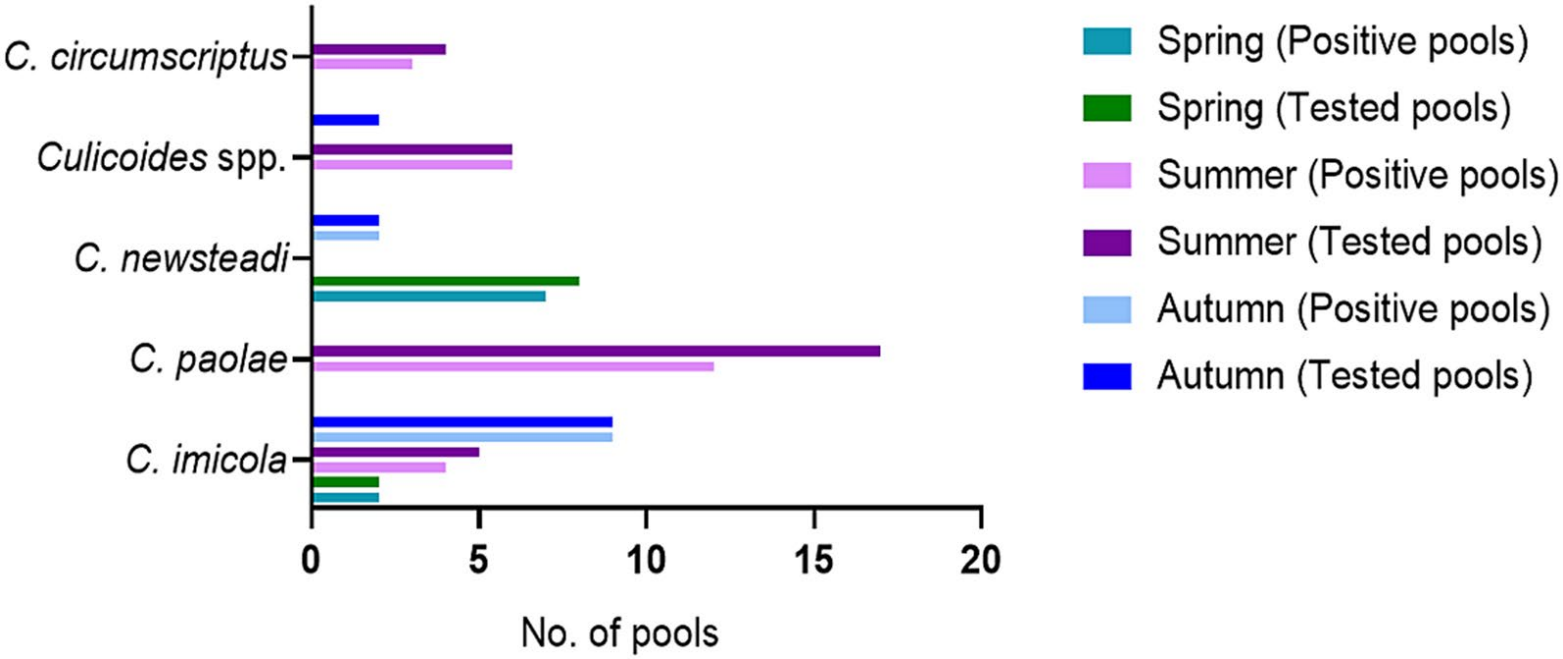
The batches of *Culicoides* species screened for bluetongue virus by RT-qPCR per season

In spring, all tested batches of *C. imicola* were positive (2/2), as were seven out of eight of *C. newsteadi*. For the summer pools, four of five *C. imicola*, 12 of 17 *C. paolae*, three of four *C. circumscriptus*, and all *Culicoides* spp. pools tested (6/6) were positive. In the autumn population, all pools of *C. imicola* (9) and *C. newsteadi* (2) were positive, while both batches of *Culicoides* spp. tested were negative (Fig. 3).

The statistical analysis revealed no significant differences among collection seasons (Chi-square test, *χ* 2 = 0.812, *df* = 2, *P* = 0.666; Fisherʼs exact test, *P* = 0.891).

### The infection rates of bluetongue virus in *Culicoides* species (MIR and MLE)

The MIR and MLE are key indicators of *Culicoides* dynamics in BTV transmission. Assuming that a PCR-positive batch includes at least one infected female, the average MIR for *Culicoides* was 2.2% (95% CI: 1.6–2.9%). However, MLE directly estimates the proportion of infected insects in the pool, giving an average value of 4.9% (95% CI: 3.3–6.4%). Both MIR and MLE were calculated to understand the distribution of bluetongue within the dominant *Culicoides* species in Morocco and across the seasons covered by the survey.

**Fig. 4:**
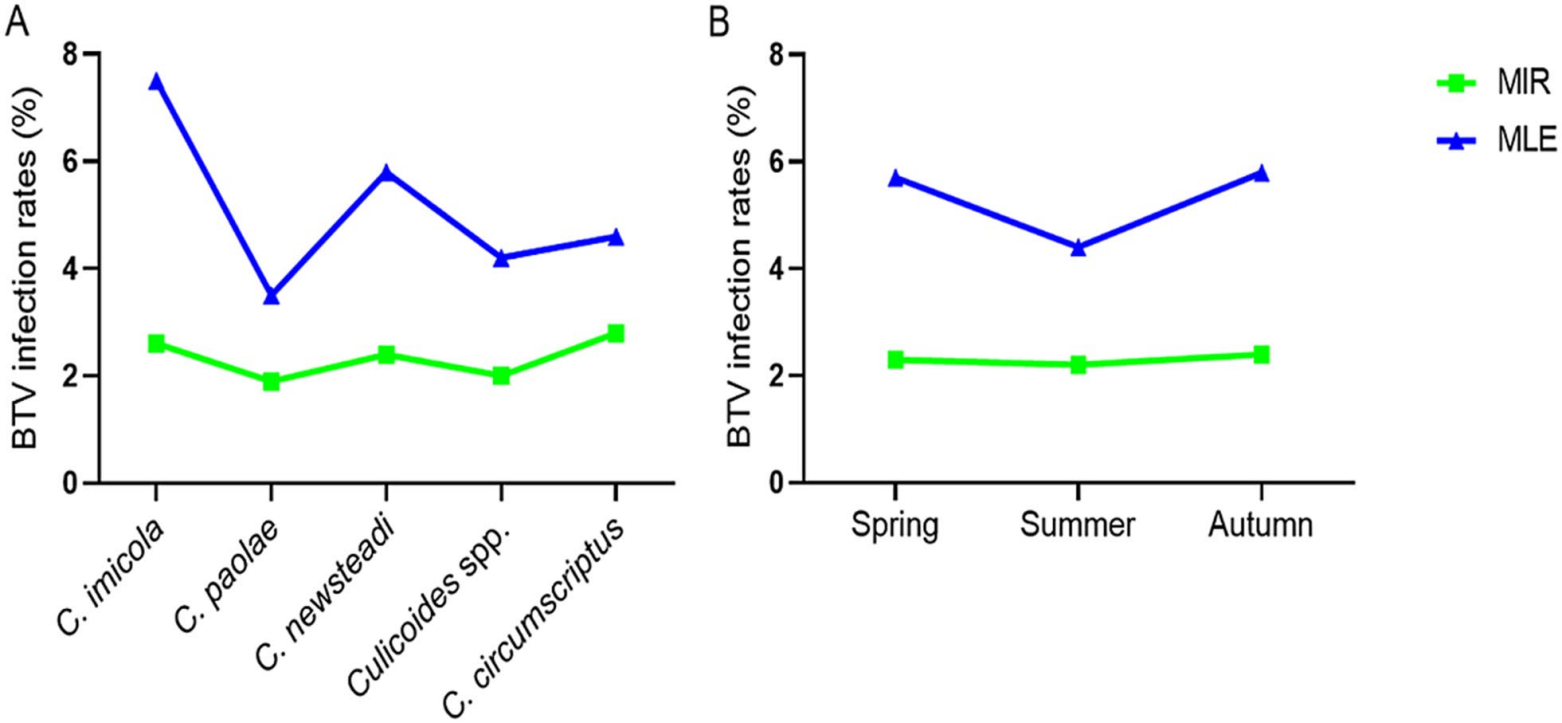
Variations in the infection rates of bluetongue virus (MIR and MLE) in *Culicoides* midges tested across species (**A**) and seasons (**B**). *BTV* bluetongue virus; *MIR* minimum infection rate; *MLE* maximum likelihood estimation

*Culicoides imicola*, *C. paolae*, *C. newsteadi*, *Culicoides* spp., and *C. circumscriptus* showed comparable MIRs, ranging from 1.9 to 2.8% (Table 2). The highest MLE rates were found in *C. imicola* (7.5%) and *C. newsteadi* (5.8%), while the other species also showed significant MLE values between 3.5 and 4.6% (Fig. 4A).

**Table 2:** Infection rates of bluetongue virus in parous *Culicoides* females captured in Morocco

Species	Tested specimens	Batch sizes	MIR in %	95% CI (%)	MLE in %	95% CI (%)
*C. imicola*	579	[30–40]	2.6	1.3–3.9	7.5	2.5–12.5
*C. paolae*	641	[16–46]	1.9	0.8–2.9	3.5	1.4–5.5
*C. newsteadi*	381	[32–40]	2.4	0.8–3.9	5.8	1.3–10.3
*Culicoides* spp.	293	[19–45]	2	0.4–3.7	4.2	0.6–7.9
*C. circumscriptus*	109	[19–34]	2.8	0–5.8	4.6	0–10.3

*MIR* minimum infection rate; *MLE* maximum likelihood estimation; *CI* confidence interval

The infection rates of *Culicoides* for three seasons of capture, spring, summer, and autumn, were similarly high (Table 3), ranging from 2.2 to 2.4% for MIR and 4.4 to 5.8% for MLE. The highest rates were reached in autumn and spring (Fig. 4B).

**Table 3:** Infection rates of bluetongue virus in parous *Culicoides* females according to collection season

Seasons	MIR in %	95% CI (%)	MLE in %	95% CI (%)
Spring	2.3	0.8–3.8	5.7	1.2–10.1
Summer	2.2	1.3–3	4.4	2.5–6.2
Autumn	2.4	1–3.9	5.8	1.9–9.7

*MIR* minimum infection rate; *MLE* maximum likelihood estimation; *CI* confidence interval

## Discussion

The parous females of *C. imicola*, *C. paolae*, *C. newsteadi*, *Culicoides* spp., and *C. circumscriptus* collected across different regions of Morocco (6 out of 12 Moroccan regions) were screened for BTV RNA using RT-qPCR. The analysis revealed BTV positivity in 45 of the 55 batches tested. The detection of the virus in the head, thorax (containing salivary glands), and abdomen with narrow Ct values (Ct < 30) for each species indicated BTV replication in the body of these species and their potential implication as BTV vectors [25, 31]. An understanding of the barriers that control the dissemination of BTV within the *Culicoides* body is essential for studying their competence and susceptibility to infection.

*Culicoides* females ingested approximately 100 TCID50 (50% tissue culture infectious dose; an average of 10^6^ TCID50/ml of virus) during a blood meal of typically 10^–4^ ml. After feeding, the viral concentration decreased following inactivation of part of the viral particles in the intestine and excretion via the anus. Subsequently, the viral load increased, reaching a plateau of around 5–6 log_10_ TCID50 a week later, corresponding to a viral multiplication of 10^3^ to 10^4^ per midge. The intestinal wall represents the first barrier—the mesenteron infection barrier (MIB)—crossed by the virus. Only the particles remaining in the intestinal lumen can infect intestinal cells. To enter the hemocoel, the virions infecting the intestinal cells must face a second barrier, the mesenteron escape barrier (MEB). Once inside the hemocoel, these viral particles contaminate secondary organs. Before reaching the salivary glands, particles infect adipocytes, representing the third barrier, the dissemination barrier (DB), which can destroy part of the virus. At higher concentrations, viral particles can successfully cross the fourth barrier, the salivary gland infection barrier (SGIB), to penetrate the salivary glands. However, for an insect to infect a new susceptible host, virus particles must also overcome the fifth and final barrier, the salivary gland escape barrier (SGEB), which depends on genetic factors [32, 33]. The interplay between the virus and the insect’s innate immune system, expressed at the barriers, plays a crucial role in the outcome of virus infection and vector survival. The primary antiviral defense mechanism involves the RNA interference (RNAi) pathway, reacting to the detection of the virus-derived double-stranded RNA to suppress its replication. Other innate immune pathways such as Toll, IMD (immune deficiency), and JAK/STAT (Janus kinase/signal transducer and activator of transcription), as well as the autophagy pathway, are also implicated in regulating immune responses in *Culicoides*. Nonetheless, the midges display refractory and permissive lineages, underlining the heritability of traits linked to their vectorial competence. This indicates that *Culicoides* exhibit inter-individual variability in vector competence, relating to their transcriptomes and the genomic characteristics of refractory and permissive lineages [21, 22, 34–37]. Accordingly, the mechanisms of virus transmission by *Culicoides* can differ according to the virus in question and *Culicoides* species. That is why certain *Culicoides* species may not express some of these barriers, as observed in *Culicoides variipennis*, which does not appear to exhibit SGIB or SGEB for BTV [23, 38]. Indeed, detecting the BTV in the head and thorax after it has crossed several barriers could indicate its successful dissemination within the *Culicoides* body [20, 23].

The calculation of MIR and MLE is essential for assessing the transmission risk of vector-borne diseases. MIR estimates the minimum number of infected vector species among a given sample of vectors, while the MLE accurately estimates the infection prevalence in the vector population. The interpretation of infection rates, especially for low abundance species with small batch sizes, can lead to abnormally high rates. Calculating infection rates using both indices, with MLE considering the size of the tested pools, helps overcome this bias [26]. These infection rates can be employed to evaluate the vectorial competence of *Culicoides* [24]. The high infection rates (≥ 1‰) recorded in field populations in the current study suggest a significant risk of BTV transmission in Morocco [39].

The findings revealed active BTV circulation during the survey seasons (spring, summer, and autumn), with increased proportions of positivity. Although the statistical difference in pool positivity between seasons and among species was not statistically significant, it suggests a similar potential for different species to transmit the virus across the three seasons. Nevertheless, the highest MIR and MLE infection rates were observed in autumn and spring. These collection seasons are associated with favorable circumstances for vector activity, thereby increasing the opportunities for BTV transmission [40, 41].

The roles of *C. imicola* and *C. newsteadi* as BTV vectors have previously been identified in the literature [25, 42–46]. Their abundance, along with their highest positivity and infectious rates, confirms, for the first time, their role as a primary potential vector of BTV in Morocco.

*Culicoides paolae* was initially implicated in BTV transmission in Sardinia in 2017, and *C. circumscriptus* has recently been found BTV-positive in Turkey and Sardinia [42, 47]. Both species were frequently collected in the current study, and their tested pools exhibited strong positivity for BTV. Higher MIR and MLE values were associated with these *Culicoides* species, indicating their greater susceptibility to BTV. As a result, this study was the first to highlight the implications of *C. paolae* and *C. circumscriptus* as potential vectors of BTV at the national level.

Meanwhile, numerous specimens of *Culicoides* spp. lacking wing spots and posing identification challenges were captured en masse in this survey. Wing spot patterns are commonly used to identify *Culicoides* species. However, *Culicoides* can also be identified based on features other than wing spots. These include microscopic details of the antennae, maxillary palps, and genitalia, as well as morphological characteristics such as body and wing size, the coloration of the dorsum of the thorax, type and number of antennal sensilla, and leg color patterns. This approach is time-consuming and impractical for a large number of individual midges. To address this limitation, molecular methods can be used for the rapid identification of *Culicoides* species [13, 29, 48–51]. Given the frequency of these *Culicoides* spp. in Morocco, RT-qPCR analysis was carried out on batches of these midges to detect BTV and assess their possible contribution to its spread in the country. The pools exhibited impressive infection rates, highlighting the potential role of these midges in BTV epidemiology. This emphasizes the need for more detailed molecular analysis to identify them, as other *Culicoides* species could be competent to transmit BTV in Morocco [48, 52–54].

Several criteria are required to confirm that an arthropod serves as an arbovirus vector. Firstly, it is necessary to assess the abundance and wide distribution of the suspected arthropod. Secondly, it is imperative to demonstrate that this arthropod has fed on a susceptible host. Furthermore, the virus must be isolated from naturally infected arthropods. It is also important to prove the ability of the arthropod to become infected by feeding either on a viremic host or on a laboratory-infected blood meal. Ultimately, the ability of the infected arthropod to transmit the virus to a susceptible host during hematophagy needs to be confirmed [55–57]. While BTV infection levels were high and the virus was well-spread in all *Culicoides* body regions, confirming the competence of the vector requires further validation through virus isolation and experimental infection studies [25].

BTV was detected in small ruminants in the same study regions during the same survey period [7], providing evidence of the active circulation of the virus and the impact of these *Culicoides* species in bluetongue outbreaks in Morocco. The diversity of BTV-vector *Culicoides* species in the Moroccan fauna poses a potential threat to livestock herds, reinforcing the importance of implementing effective management strategies and vector control programs.

## Conclusions

The current study provided the first molecular evidence illustrating the involvement of *C. imicola*, *C. paolae*, *C. newsteadi*, *Culicoides* spp., and *C. circumscriptus* as potential vectors for BTV propagation at the national level, along with insights into the vector activity seasons of these insects. BTV detection in parous females of field species, after the virus has crossed multiple body barriers, and the analysis of infection rates (MIR and MLE) for these midge populations served to assess the vectorial competence of *Culicoides*. Indeed, the results confirmed the implication of the studied species (*C. imicola*, *C. paolae*, *C. newsteadi*, *Culicoides* spp., and *C. circumscriptus*) as potential BTV vectors in bluetongue epidemics in Morocco, with active virus circulation during spring, summer, and autumn.

## Data Availability

All data generated or analyzed during this study are included in this published article.

## Abbreviations

BTV: Bluetongue virus
OVI: Onderstepoort Veterinary Institute
RT-PCR: Reverse transcription polymerase chain reaction
MLE: Maximum likelihood estimation
MIR: Minimum infection rate
WOAH: World Organization for Animal Health
EDTA: Ethylenediaminetetraacetic acid
PBS: Phosphate-buffered saline
RT-qPCR: Reverse transcription quantitative polymerase chain reaction
Ct: Cycle threshold
CI: Confidence interval
TCID50: 50% Tissue culture infectious dose
MIB: Mesenteron infection barrier
MEB: Mesenteron escape barrier
DB: Dissemination barrier
SGIB: Salivary gland infection barrier
SGEB: Salivary gland escape barrier
RNAi: RNA interference
IMD: Immune deficiency
JAK/STAT: Janus kinase/signal transducer and activator of transcription
